# Negative Effects On Cancer Care Due To COVID-19 Implications: Observations From A Cancer Center In The UAE

**DOI:** 10.7150/jca.51792

**Published:** 2020-09-24

**Authors:** Shaheenah Dawood, Zulfaqqar Jaffar, Zakir K Mohamed, Annett Al-Hamadi, Zakaullah Khan

**Affiliations:** 1 Department of Medical Oncology, Mediclinic City Hospital, Mohammed Bin Rashid University of Medicine and Health Sciences, Dubai, UAE; 2 Department of Medical Oncology, Mediclinic Abu Dhabi, UAE; 3 Department of GI Surgery, Mediclinic Parkview, Dubai, UAE; 4 Department of Breast Surgery, Mediclinic City Hospital, Dubai, UAE; 5 Mediclinic Health Care, Dubai, UAE

In 2019, the World Health Organization declared the novel coronavirus disease (COVID-19) to be a public health emergency of international concern that rapidly escalated to pandemic status in 2020 1. As of August 2, 2020, there have been approximately 17.6 million confirmed cases worldwide, with over 680,000 deaths from this disease 1. A particularly vulnerable cohort due to immunosuppression are those that suffer from cancer. On the 14^th^ of February 2020 a nationwide analysis was published of patients suffering from COVID-19 in China that indicated that 18 of 1590 patients (1%) had a history of cancer and had higher risk of respiratory complications, more time spent in the intensive care unit, and a shorter time to deterioration compared to patients who did not have cancer 2. On the 28^th^ of May 2020 we saw the results of the COVID-19 and Cancer Consortium (CCC19) database that reported a 30 day all-cause mortality of 13 % among patients with either active or previous history of cancer who had contracted COVID-19 with higher rates observed among those who were older, had poor performance status or were receiving active cancer related therapy 3. Various groups have made recommendations that include modifications in adjuvant therapy, delay in surgery and chemotherapy if feasible, and de-prioritizing patients with metastatic cancer and delayed screening strategies 4. Although these recommendations were sensible in light of the pandemic, the clinical care pathways for patients with cancer that have been built over the years in an effort to ensure early detection and therapy have been severely disrupted. As we pass the peak of the pandemic, the negative impact of our decisions will now become more visible.

## The UAE Experience: Building a cancer fortress

Our group runs one of the largest comprehensive cancer centers in the UAE that caters to approximately 20% of the 9.8 million of the national population. Recognizing the need to be able to protect patients adequately was paramount, without compromising their care in either the adjuvant or metastatic setting - which could cause long term negative consequences. In addition to the general recommendations of screening patients for fever and symptoms related to COVID-19 and minimizing clinic visits by actively adopting telemedicine consultations, we were in a unique position to mandate COVID-19 screening for all patients with cancer who were actively getting chemotherapy, targeted therapy, immunotherapy and or radiation therapy, at baseline and at the start of each cycle, throughout the course of their treatment. For patients on regular follow up after completion of all therapy, COVID-19 testing was only required in the presence of symptoms or history of exposure. COVID-19 screening was also mandated prior to any cancer-related surgery. COVID-19 testing was done by Reverse Transcriptase PCR assay on specimens derived from a nasopharyngeal swab. Between March 20^th^ and May 7^th^ of 2020, 353 patients with current or prior history of cancer were screened with the majority (83.6%) complying with the protocol mandate**.** Of the 295 patients tested, fourteen (4.7%) tested positive, representing 0.5% of all patients who tested positive with and without a history of cancer (n=2685) at our institution. Eight (57.1%) of those 14 patients were actively receiving or had recently completed therapy **(Table 1)**. Four patients did not have symptoms, one of whom tested positive prior to breast surgery, while one patient actively receiving chemotherapy for ovarian cancer had recent travel history. One patient with breast cancer receiving neoadjuvant chemotherapy, who tested positive for COVID-19 after exposure to a positive family member, required time in the intensive care unit and was successfully treated with plasma exchange therapy. Two patients (15.4%) who initially tested negative were re-tested and found to be positive indicating that the true prevalence may be higher than that reported due to the false negative results for tests to detect COVID-19 5. One patient (7%) with metastatic advanced colorectal cancer on immunotherapy who had failed multiple lines of therapy died in the intensive cancer unit after testing positive for COVID-19. Increased and early use of GCSF support and antibiotics was also observed.

## The Dilemma we now face

Routine testing of all patients with cancer who are actively receiving therapy provided physicians with more confidence to adhere to guideline-based protocols as highlighted by minimal protocol violation observed among patients who were actively being treated, with therapy delivered largely on schedule. Our experience also reflects the results of the recent prospective observational study from the UK Coronavirus Cancer Monitoring Project (UKCCMP) that patients with cancer receiving anti-cancer therapy did not appear to be at higher risk of mortality from COVID-19 6. We were confident that we were treating our patients well and we were not going to negatively impact long term prognostic outcome. However, as we entered the plateau in our pandemic curve and services opened up, we observed four very disturbing facts. First, a number of patients who were on active anti-cancer therapy, most with curative intent, abandoned therapy during the peak of the pandemic due to fear of COVID-19 exposure. Second, during the peak of the pandemic, elective surgeries including a number of cancer related surgeries had to be postponed with patients now returning with evidence of locoregional progression and distant metastatic disease. Third, screening programs (a critical first step in the cancer care pathway) including those for breast and GI had seen a sharp dip during the pandemic due either to closure or restriction of services to the most urgent cases. As such now that that these services had re opened, we were picking up more advanced cases. Fourth, patients with symptoms such as breast lumps or rectal bleeding were simply too scared during the peak of the pandemic to seek medical advice and evaluation and were now presenting with more advanced stages of disease. In an epidemiological study deriving data from weekly diagnostic referral and chemotherapy treatments until April 2020 in England, an estimated 20% increase in cancer mortality has been predicted due to delays in either diagnosis or treatment administration due to the pandemic 7.

## Next steps forward

Multiple guidelines have been published in 2020 highlighting the need to consider modifications to treatment regimens based on the current pandemic we are facing. The guidelines are not based on definitive data but rather on consensus opinion. The drawback is that we may impact long term prognostic outcome negatively albeit inadvertently, in an attempt to decrease risk of acquiring COVID-19. Intensification of testing strategies, in addition to symptom-screening strategies and minimization of clinic visits for patients with history of cancer, can aid and inform early intervention strategies, to treat this vulnerable group and reduce associated morbidity and mortality significantly. However, these guidelines have not addressed the chaos that the pandemic has thrown health care workers and patients into. The years of intensive screening programs and patient education that has been effective in decreasing mortality rates associated with cancer has just taken a major step backwards. At our center we have taken active measures to decrease lag time associated with consultations, screening and diagnostics, and enhance patient confidence to get back to seeking these services by ensuring that the environment for these procedures is safe with active cleaning protocols and COVID-19 testing protocols in place for both patients and associated health care providers. Is this going to stop the rise of advanced cancer cases that we anticipate? Perhaps, but not completely. It is however an important first step in treating patients as quickly and efficiently as we can. We are going to have to work as a team to proactively reformulate our strategy moving forward - the storm may have passed; a tsunami is about to arrive.

## Figures and Tables

**Table 1:**
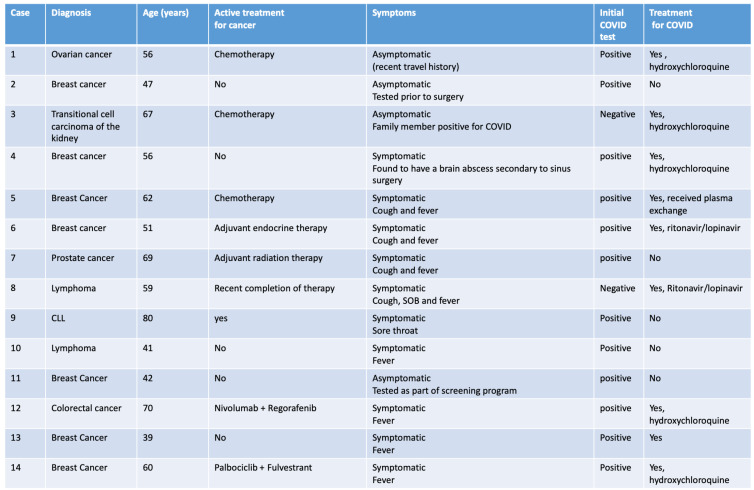
Characteristics of COVID positive patients with recent or prior history of Cancer (N=14)
